# The Effectiveness of a Web-Based Computer-Tailored Physical Activity Intervention Using Fitbit Activity Trackers: Randomized Trial

**DOI:** 10.2196/11321

**Published:** 2018-12-18

**Authors:** Corneel Vandelanotte, Mitch J Duncan, Carol A Maher, Stephanie Schoeppe, Amanda L Rebar, Deborah A Power, Camille E Short, Christopher M Doran, Melanie J Hayman, Stephanie J Alley

**Affiliations:** 1 Physical Activity Research Group, Appleton Institute School of Health, Medical and Applied Sciences Central Queensland University Rockhampton Australia; 2 School of Medicine & Public Health Faculty of Health and Medicine The University of Newcastle Newcastle Australia; 3 School of Health Sciences, Alliance for Research in Exercise, Nutrition and Activity University of South Australia Adelaide Australia; 4 School of Health, Medical and Applied Sciences Central Queensland University Rockhampton Australia; 5 School of Medicine, Freemasons Foundation Centre for Men's Health University of Adelaide Adelaide Australia

**Keywords:** online, internet, tracking, health behavior change, advanced activity trackers, wearables

## Abstract

**Background:**

Web-based interventions that provide personalized physical activity advice have demonstrated good effectiveness but rely on self-reported measures of physical activity, which are prone to overreporting, potentially reducing the accuracy and effectiveness of the advice provided.

**Objective:**

This study aimed to examine whether the effectiveness of a Web-based computer-tailored intervention could be improved by integrating Fitbit activity trackers.

**Methods:**

Participants received the 3-month *TaylorActive* intervention, which included 8 modules of theory-based, personally tailored physical activity advice and action planning. Participants were randomized to receive the same intervention either with or without Fitbit tracker integration. All intervention materials were delivered on the Web, and there was no face-to-face contact at any time point. Changes in physical activity (Active Australia Survey), sitting time (Workforce Sitting Questionnaire), and body mass index (BMI) were assessed 1 and 3 months post baseline. Advice acceptability, website usability, and module completion were also assessed.

**Results:**

A total of 243 Australian adults participated. Linear mixed model analyses showed a significant increase in total weekly physical activity (adjusted mean increase=163.2; 95% CI 52.0-274.5; *P*=.004) and moderate-to-vigorous physical activity (adjusted mean increase=78.6; 95% CI 24.4-131.9; *P*=.004) in the Fitbit group compared with the non-Fitbit group at the 3-month follow-up. The sitting time and BMI decreased more in the Fitbit group, but no significant group × time interaction effects were found. The physical activity advice acceptability and the website usability were consistently rated higher by participants in the Fitbit group. Non-Fitbit group participants completed 2.9 (SD 2.5) modules, and Fitbit group participants completed 4.4 (SD 3.1) modules.

**Conclusions:**

Integrating physical activity trackers into a Web-based computer-tailored intervention significantly increased intervention effectiveness.

**Trial Registration:**

Australian New Zealand Clinical Trials Registry ACTRN12616001555448; https://www.anzctr.org.au/Trial/Registration/TrialReview.aspx?id=371793 (Archived by WebCite at http://www.webcitation.org/73ioTxQX2)

## Introduction

### Background

Regular physical activity is recommended to reduce the risk of developing chronic disease (eg, diabetes, cardiovascular disease, and cancer), mental health problems, mortality, and morbidity [1,2]. Unfortunately, in Australia, and in most other developed and developing nations, the majority of the population is not meeting the physical activity recommendations [1,3]. This causes a large burden of disease, reduced quality of life, and high health care costs [2,4]. As such, the search for cost-effective interventions that can effectively increase physical activity levels in large populations is ongoing [5].

In this regard, Web-based computer-tailored interventions have demonstrated promising outcomes. Computer-tailored interventions aim to mimic face-to-face interactions with health professionals and provide highly detailed and personally relevant behavior change information [6,7]. However, unlike face-to-face experience, they have a wide reach with access to unlimited numbers of Web users at low cost [6,7]. Personalized physical activity advice is provided after participants complete 1 or more Web-based surveys. On the basis of participant responses and using IF-THEN algorithms (eg, IF not meeting activity guideline, THEN provide advice to increase activity levels), relevant feedback is selected from a large database with all possible response options [8]. Although a systematic review found that 80% of studies that provided Web-based personalized physical activity advice reported positive results at 3 months, the effect sizes were relatively small and less than half of the studies (47%) found significant effects 6 months after starting the study, meaning that intervention effects are not maintained [6].

As such, there is scope to improve the effectiveness of computer-tailored interventions. An important limitation is that they depend on Web-based self-report physical activity measures to generate personalized advice. It is well known that many people overestimate their self-reported activity levels by a large margin [9]. For example, an Australian study showed that 24% of the general population (and up to 58% in certain subgroups) overreported their activity levels [9]. Inaccurate self-reported physical activity can lead to participants being provided with incorrect advice [10]. For example, because of overreporting, someone might receive the message that they are meeting the activity guidelines and do not need to become more active, when this is actually not the case. When this happens, the intervention is not providing accurate and credible advice to participants and will, therefore, not be as effective as it could be [10,11]. Therefore, new techniques to increase the effectiveness of computer-tailored interventions are needed.

The proliferation of sophisticated activity trackers (eg, Fitbit) provides a unique opportunity to improve the effectiveness of computer-tailored interventions. These advanced activity trackers can measure steps, heart rate, energy expenditure, sleep, sedentary behavior, and physical activity intensity (ie, light, moderate, or vigorous intensity) [12]. Furthermore, they allow for automated data uploads to websites or apps via a wireless connection. As such, these activity trackers can objectively and accurately assess physical activity through continuous monitoring [13]. The data generated by these activity trackers can then conveniently and seamlessly be integrated into computer-tailored advice without the burden of repeated Web-based surveys, thus increasing the potential for providing computer-tailored advice that is more credible and effective when compared with using less reliable self-reports [11]. Moreover, replacing the Web-based surveys by activity trackers may lead to greater intervention adherence, as participants in previous computer-tailored studies have systematically reported that there are too many questions that need to be answered before they receive their personalized advice [14,15].

### Objectives

Therefore, the objective of this 2-group randomized trial was to examine whether a Web-based computer-tailored intervention using Fitbit activity trackers to generate personalized feedback is more effective in increasing physical activity and engaging participants compared with a computer-tailored intervention using traditional self-reports.

## Methods

### Procedures and Participants

Participants were recruited across Australia using random digit dialing (conducted by the Population Research Lab at Central Queensland University [CQUniversity]), Facebook advertisements, flyers, posters, word-of-mouth, and email lists (ie, people who signed up to the Web-based 10,000 Steps program [only those who had not used the program for at least 12 months were invited], CQUniversity alumni). Those interested were directed to a landing page on the intervention website to complete a screening tool that determined eligibility. Eligible participants were aged 18 years or above, living in Australia, had a smartphone and computer with internet access, scored 2 or more out of 5 on the Internet Self-Confidence Scale [16], able to speak and read English, had a body mass index (BMI) between 25 and 40, engaged in less than 150 min per week of moderate-to-vigorous physical activity [17,18], had no prior experience in using an activity tracker, had not participated in a physical activity intervention within the last 12 months, and were able to safely increase physical activity assessed through the physical activity readiness questionnaire (PAR-Q) [19]. Those not meeting PAR-Q standards were instructed to obtain medical clearance before participation was allowed.

After completing the Web-based screening tool, eligible participants completed Web-based baseline surveys (see Measures section below). After completing baseline assessments, participants were randomized into 1 of the 2 groups in a ratio of 1:1 using a random list generator and provided with access to the *TaylorActive* intervention (see Intervention section below). All participants received access to the *TaylorActive* intervention; however, only 1 group (the *Fitbit* group) received a Fitbit activity tracker to monitor physical activity objectively, and the other group (the *Non-Fitbit* group) did not. In the *Fitbit* group, participants were posted a Fitbit Flex, along with instructions on how to use it and sync data from the Fitbit to the *TaylorActive* website. They only received access to the *TaylorActive* intervention 7 days following receipt of the Fitbit, so that it could collect physical activity data that could then be immediately synced with the *TaylorActive* website upon first use. Access was not delayed in the non-Fitbit group, as participants were able to self-report the last week of activity immediately. Follow-up measures were assessed 1 and 3 months post baseline. Participants in both groups received up to 3 reminder emails and 2 phone calls/text messages when they did not complete the surveys within the desired time frame. There was no face-to-face contact with participants throughout the entire duration of this study; all procedures were Web-based, via phone or postal mail. Participants who complied with all study procedures received an Aus $50 incentive for their participation; those in the Fitbit group were able to decline the incentive in exchange for keeping the Fitbit they received (they were only informed about this option at the end of the study).

All participants provided informed consent, ethical approval was obtained from the CQUniversity Human Ethics Committee (H1608-227), and the trial was registered at the Australian New Zealand Clinical Trails Registry (ACTRN12616001555448). All data were collected and analyzed in 2016 and 2017.

### Intervention

Participants in both groups received access to a computer- tailored physical activity intervention named *TaylorActive* [20]. The behavior change content of this intervention was developed in line with the theory of planned behavior [21], self-determination theory [22], and social cognitive theory [23]. Specifically, content was focused on enhancing intrinsic motivation, self-efficacy, and intentions for increasing activity levels. In addition, training was provided on self-regulatory strategies to enhance the enactment of intentions into behavior through effective goal-setting, action planning, use of social support, overcoming barriers, problem solving, decision making, relapse prevention, and self-monitoring [21-23].

On the basis of short Web-based surveys, participants in both groups were provided with behavior change content across 8 modules of personal physical activity advice delivered over a 3-month period. The first 4 modules were delivered weekly; the next 4 modules were delivered every 14 days. The 8 modules were organized in a set order and the *next* module could only be accessed when the previous module was completed. All modules were released at a set time point based on participants’ study start date. If participants did not access newly available modules, they received up to 3 reminder emails. To generate the personalized module content in the non-Fitbit group, participants were asked questions about how active they have been the previous week in conjunction with questions relating to individual, social, environmental, and theory-based correlates of physical activity behavior. On the basis of the answers of participants, and through applying IF-THEN algorithms, personally relevant physical activity content was automatically selected from a database. In the first session, participants were asked to select their preference of 1 of 5 motivations to be physically active: (1) to improve or maintain good health, (2) to increase fitness, (3) to increase strength, (4) to lose weight, or (5) to feel better (improve mood and/or reduce stress). The feedback and physical activity goals were tailored according to participants’ preferred motivation.

The only difference between groups was the way in which physical activity was assessed to provide personalized advice for the 8 modules. In the *non-Fitbit* group, participants completed an adapted version of the *Godin-Shephard Leisure-time Exercise Questionnaire* at the start of each module [24]. In the *Fitbit* group, physical activity was assessed using a Fitbit Flex (this device does not have a display other than 5 tiny LEDs; 1 LED illuminates for every 2000 steps taken; this device does not nudge or buzz or beep when participants have not been active for a while). Participants only needed to click 1 button on the *TaylorActive* website at the start of each module to import physical activity data collected using the Fitbit. The physical activity advice was structured in the same way for both groups, as equivalent variables were extracted from both assessment methods (light, moderate, vigorous, and total physical activity).

Participants in both groups also had access to a *Library* with generic educational information about physical activity; a total of 19 brief articles were available about different aspects of physical activity and what to do to increase physical activity levels (eg, “Are you physically fit?,” “Getting motivated,” and “Making time to be active”). Finally, participants in both groups were encouraged to complete an action plan at the end of each module [20]. Action plans are self-regulation strategies in the form of setting up a detailed plan that can lead to better goal attainment and help in behavior modification [25]. Practically, it meant that participants were asked very specific questions on how they would meet their activity goals: what activity they would do, where they would do it, when they would do it, how often they would do it, how long will each activity session be, and with whom they would do it. At the start of creating an action plan, participants were asked to set long-, medium-, and short-term goals to reach their physical activity objectives.

More in-depth details about this intervention can be found in the protocol paper for a different trial, only the “Intervention” section (starting on page 3) from that paper is relevant for the study described here [20]. As outlined in this protocol paper, there are in fact 2 versions of the *TaylorActive* intervention, 1 version in which all personalized feedback is provided as text on a webpage and the other version where feedback is delivered through personalized videos. As the main *TaylorActive* trial is still ongoing, it was unknown at the time of this study which version was more effective. As such, participants in this study were equally randomized to text and video versions. Any effects caused from these different versions were controlled for in the statistical analysis. Discussing the impact of the different versions of the *TaylorActive* intervention is outside the scope of this paper.

### Measures

Basic demographic factors were assessed: sex, age, years of education, income (≤Aus $51,999; Aus $52,000-Aus $99,999; ≥Aus $100,000; don’t know or no response), employment status (full-time, part-time or casual, unemployed), height (centimeters), and weight (kilograms). Height and weight measures were used to calculate BMI of participants.

The 8-item *Active Australia Survey* was used to measure changes in physical activity (please note: the Godin-Shephard Leisure-time Exercise Questionnaire was only used to provide participants in the non-Fitbit group with personalized activity advice; it was not used to assess study outcomes). This survey assesses frequency and duration of walking for transport, walking for recreation, moderate intensity physical activity, and vigorous intensity physical activity [26]. Total physical activity was calculated by summing the time spent in walking, moderate activity, and vigorous activity (weighted by 2) according to specified scoring guidelines [26]. Moderate-to-vigorous physical activity was also calculated and did not include the time spent walking. The Active Australia Survey has acceptable test-retest reliability (intraclass coefficient=.64) and validity (*r*=.61) in the Australian adult population and has been documented as a useful evaluative tool for detecting intervention-related change in physical activity [27,28].

Sitting time was measured using the 10-item *Workforce Sitting Questionnaire* [29]. Participants reported time (hours or minutes) spent sitting on usual working and nonworking days in relation to work, transport, television use, computer use, and other leisure time sitting. One question also assessed the number of days participants usually work in a week. Total sitting time was defined as the sum of sitting time in all domains for all days. This questionnaire has demonstrated adequate test-retest reliability and validity [29].

The acceptability of the physical activity advice, website usability, and Fitbit use were also assessed [14]. These questions were based on previously published work where advice acceptability of similar interventions was assessed [14]. Finally, module completion was tracked objectively through the intervention website.

### Statistics

Analyses were conducted using SPSS version 24. Descriptive statistics of participants’ demographics, total physical activity, moderate-to-vigorous physical activity, total sitting time, and BMI at baseline are presented. Group (Fitbit and non-Fitbit) comparisons were conducted using *t* tests for continuous variables and chi-square analyses for categorical variables. To test for a group (Fitbit or non-Fitbit) by time (baseline, 1 month, and 3 months) interaction on total weekly physical activity, a linear mixed model analysis was conducted. In total, 3 more separate linear mixed model analyses were conducted to test a group by time interaction effects on moderate-to-vigorous physical activity, sitting time, and BMI. All linear mixed model analyses applied restricted maximum likelihood estimation to reduce risk of bias from missing data [30]. All linear mixed model analyses were adjusted for age, sex, education, employment, income, version of the *TaylorActive* intervention (video or text), and BMI (with exception of the model what was examining BMI itself). The non-Fitbit group was the reference variable for group, and baseline was the reference variable for time.

## Results

**Figure 1:**
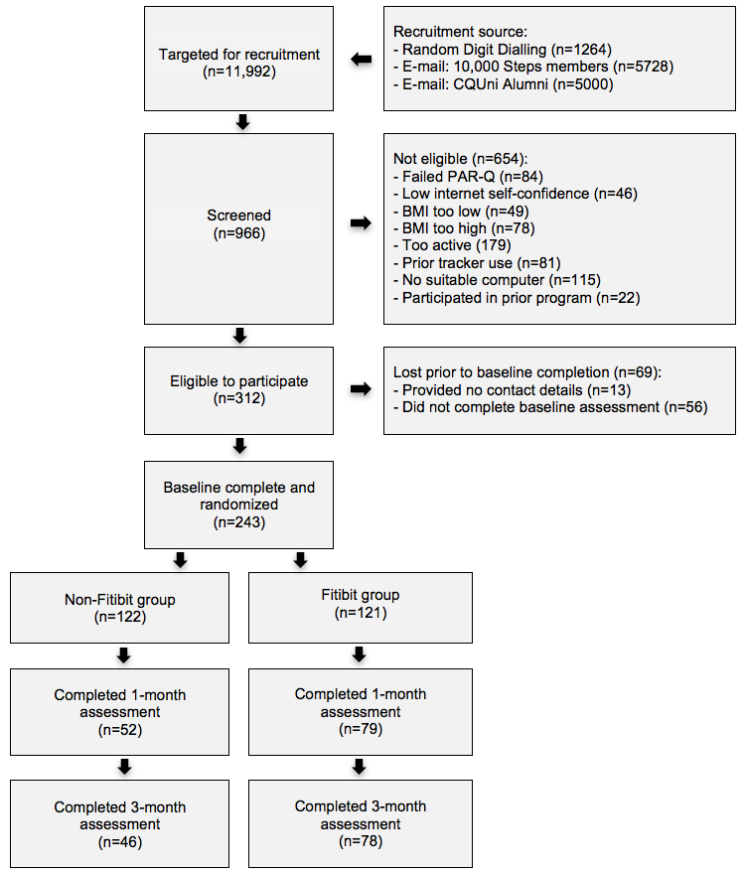
Participant flowchart. CQUniversity: Central Queensland University, PAR-Q: physical activity readiness questionnaire, BMI: body mass index.

A total of 243 participants were randomized (see Figure 1 for participant flow). The majority of participants were female (182/243, 74.9%), employed full-time (129/243, 53.1%), and earned a yearly income over Aus $51,000 (179/243, 61.0%). The average age, BMI, and years of education were 51.5, 31.2, and 14.8, respectively. At baseline, participants engaged in 106.8 min per week of total physical activity and 36.6 min per week of moderate-to-vigorous physical activity; average daily sitting time was 10 hours a day. There were no between-group differences at baseline. Significantly more participants in the non-Fitbit group did not complete assessments at 1 month (57% vs 35%; χ^2^_1_=12.5; *P*<.001) and at 3 months (63% vs 36%; χ^2^_1_=17.4; *P*<.001) compared with the Fitbit group. Participant characteristics are reported in Table 1.

**Figure 2:**
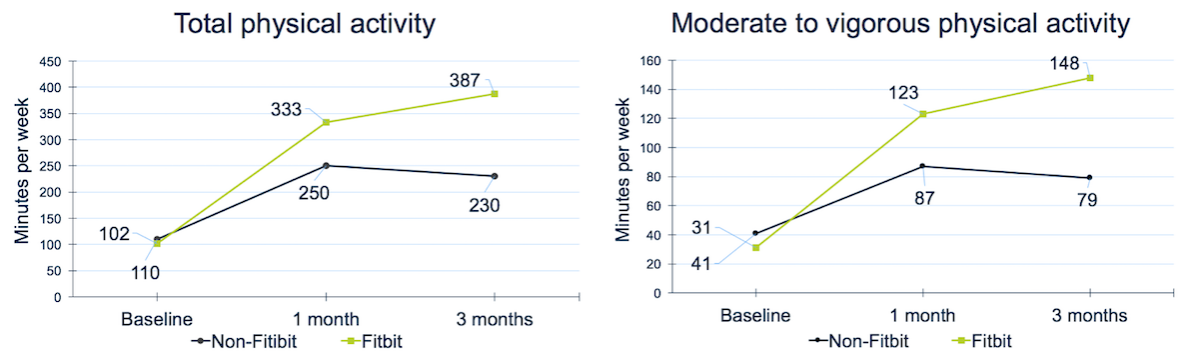
Total physical activity and moderate-to-vigorous physical activity at baseline, 1 month and 3 months.

There were significant time effects at 1 and 3 months for both groups for total physical activity and also a significant time by group interaction at 3 months (adjusted mean difference=163.2 min; 95% CI 52.0-274.5; *P*=.004) though not at 1 month (see Table 2 and Figure 2). Total physical activity increased 119.3 min per week in the non-Fitbit group and 284.9 min per week in the Fitbit group at 3 months. Similarly, significant time effects were observed at 1 and 3 months for moderate-to-vigorous physical activity as well as a significant time by group interaction at 3 months (adjusted mean difference=78.6 min; 95% CI 24.4-131.9; *P*=.004) but again not at 1 month. Total moderate-to-vigorous physical activity increased 38.3 min per week in the non-Fitbit group and 117.2 min per week in the Fitbit group at 3 months. Although there was a significant time effect for sitting time in the Fitbit group at 3 months, no other statistically significant time effects or interaction effects were found. Sitting was, on average, reduced by 56 min per day in the non-Fitbit group and 101 min per day in the Fitbit group at 3 months. For BMI, significant time effects were found at both time points for the non-Fitbit group but only at 3 months for the Fitbit group; no interaction effects were observed. BMI was reduced by 1.07 in the non-Fitbit group and 1.54 in the Fitbit group.

**Table 1:** Baseline participant characteristics as well as physical activity, moderate-to-vigorous physical activity, sitting time, and body mass index at all time points.

Baseline characteristics		All participants (N=243)	Non-Fitbit (n=122)	Fitbit (n=121)	*P* value^a^
**Sex, n (%)**					
	Male	61 (25.1)	29 (23.8)	32 (26.4)	.63
	Female	182 (74.9)	93 (76.2)	89 (73.6)	—^b^
Age in years, mean (SD)		51.5 (11.1)	51.5 (10.6)	51.6 (11.6)	.94
Education in years, mean (SD)		14.8 (3.4)	14.4 (3.0)	15.1 (3.7)	.09
**Employment, n (%)**					
	Full time	129 (53.1)	67 (54.9)	62 (51.2)	.33
	Part-time or casual	54 (22.2)	30 (24.6)	24 (19.8)	—
	Other	60 (24.7)	25 (20.5)	35 (29.0)	—
**Income, n (%)**					
	≤Aus $51,999	64 (26.3)	33 (27.0)	31 (25.6)	.14
	Aus $52,000-Aus $99,999	81 (33.3)	46 (37.7)	35 (28.9)	—
	≥Aus $100,000	67 (27.6)	33 (27.0)	34 (28.1)	—
	Don’t know or no response	31 (12.8)	10 (8.2)	21 (17.4)	—
**Recruitment source, n (%)**					
	10,000 steps database	79 (32.5)	41 (33.6)	38 (31.4)	.54
	Population research lab	79 (32.5)	41 (33.6)	38 (31.4)	—
	Facebook ads	28 (11.5)	16 (13.1)	12 (9.9)	—
	Central Qqueensland University alumni database	17 (7.0)	6 (4.9)	11 (9.1)	—
	Other	40 (16.4)	18 (14.8)	22 (18.1)	—
**Body mass index, mean (SD)**					
	At baseline	31.2 (4.5)	31.1 (4.7)	31.4 (4.4)	.63
	At 1 month	30.6 (4.3)	30.4 (4.5)	30.7 (4.2)	—
	At 3 months	30.0 (4.5)	30.1 (4.6)	29.9 (4.4)	—
**Total physical activity in minutes per week, mean (SD)**					
	At baseline	106.8 (147.4)	110.7 (150.7)	102.8 (144.4)	.67
	At 1 month	300.1 (306.4)	250.2 (293.4)	333.0 (312.1)	—
	At 3 months	329.2 (324.0)	230.0 (164.1)	387.7 (377.7)	—
**Moderate-to-vigorous physical activity in minutes per week, mean (SD)**					
	At baseline	36.6 (76.5)	41.5 (80.4)	31.6 (72.4)	.31
	At 1 month	109.3 (164.8)	87.3 (146.5)	123.8 (175.2)	—
	At 3 months	123.2 (154.3)	79.8 (77.1)	148.8 (181.1)	—
**Total sitting time in hours per day, mean (SD)**					
	At baseline	10.0 (3.6)	10.1 (3.3)	9.9 (3.8)	.59
	At 1 month	9.3 (3.7)	9.2 (3.5)	9.3 (3.9)	—
	At 3 months	8.6 (4.2)	9.2 (3.6)	8.2 (4.5)	—

^a^The *P* values reported are the outcomes of *t* tests (continuous variables) or chi-square tests (categorical variables) and only relate to comparing Fitbit and non-Fitbit groups at baseline (hence, no *P* values are reported for 1- and 3-month outcomes).

^b^Not applicable.

**Table 2:** Linear mixed models analysis comparing change in total physical activity, moderate-to-vigorous physical activity, sitting time, and body mass index between Fitbit and non-Fitbit groups at 1 and 3 months adjusted for baseline levels.

Characteristics^a^		Time-effects				Time by group interaction-effects (reference=non-Fitbit group)	
		Fitbit group		Non-Fitbit group		Adjusted mean difference from baseline (95% CI)	*P* value
		Adjusted mean difference from baseline (95% CI)	*P* value	Adjusted mean difference from baseline (95% CI)	*P* value		
**Total physical activity (weekly minutes)**							
	1 month	222.93 (154.98 to 290.87)	<.001	152.00 (80.04 to 223.96)	<.001	77.89 (−23.30 to 179.07)	.13
	3 months	270.12 (188.86 to 351.36)	<.001	110.24 (56.39 to 164.10)	<.001	163.26 (52.03 to 274.50)	.004
**Moderate-to-vigorous physical activity (weekly minutes)**							
	1 month	89.59 (50.64 to 128.53)	<.001	50.90 (19.21 to 82.60)	.002	38.37 (−16.02 to 92.77)	.17
	3 months	110.46 (72.38 to 148.54)	<.001	31.13 (5.02 to 57.24)	.002	78.65 (25.40 to 131.89)	.004
**Sitting (daily minutes)**							
	1 month	−34.33 (−88.61 to 19.94)	.21	−40.20 (−99.38 to 18.98)	.18	8.58 (−71.8 to 88.98)	.83
	3 months	−103.72 (−156.68 to −50.75)	<.001	−31.90 (−83.32 to 19.51)	.22	−70.10 (−147.74 to 7.53)	.08
**Body mass index**							
	1 month	−0.20 (−0.41 to 0.01)	.06	−0.44 (−0.72 to −0.16)	.002	0.23 (−0.12 to 0.57)	.18
	3 months	−0.72 (−1.04 to 0.40)	<.001	−0.62 (−1.03 to 0.21)	.004	−0.12 (−0.63 to 0.40)	.66

^a^Linear mixed models included all participants at all time points, as such N=243 for all analyses. Analyses were adjusted for age, gender, education, employment status, income, body mass index (BMI; the BMI model was not adjusted for BMI), and video or text advice. The reference variable for time was the baseline measure, and the reference variable for group was the non-Fitbit group.

Table 3 presents outcomes on user acceptability of the advice, intervention website, and Fitbit. Both the physical activity advice acceptability and the website usability were consistently rated higher (though not always significantly higher) by participants in the Fitbit group. In terms of advice acceptability, significant differences were found for the questions *there were too many questions to access the advice,* *the advice taught me something new about my physical activity,* and *I shared the advice with others.* In terms of website usability, significant differences were found for the questions *I want to continue to use the website,* *the website is easy to use,* and *I used the website once per week or more.* The Fitbit group also indicated the use of the Fitbit itself was favorable and augmented the personal advice delivered through the website. For example, participants indicated (*agreed* or *strongly agreed*) that the value of the tailored advice was increased (74.4%), that the advice was more credible (67.9%), and more personally relevant (76.9%). The majority of participants (85.9%) thought it was easy to sync Fitbit data with the TaylorActive website.

**Figure 3:**
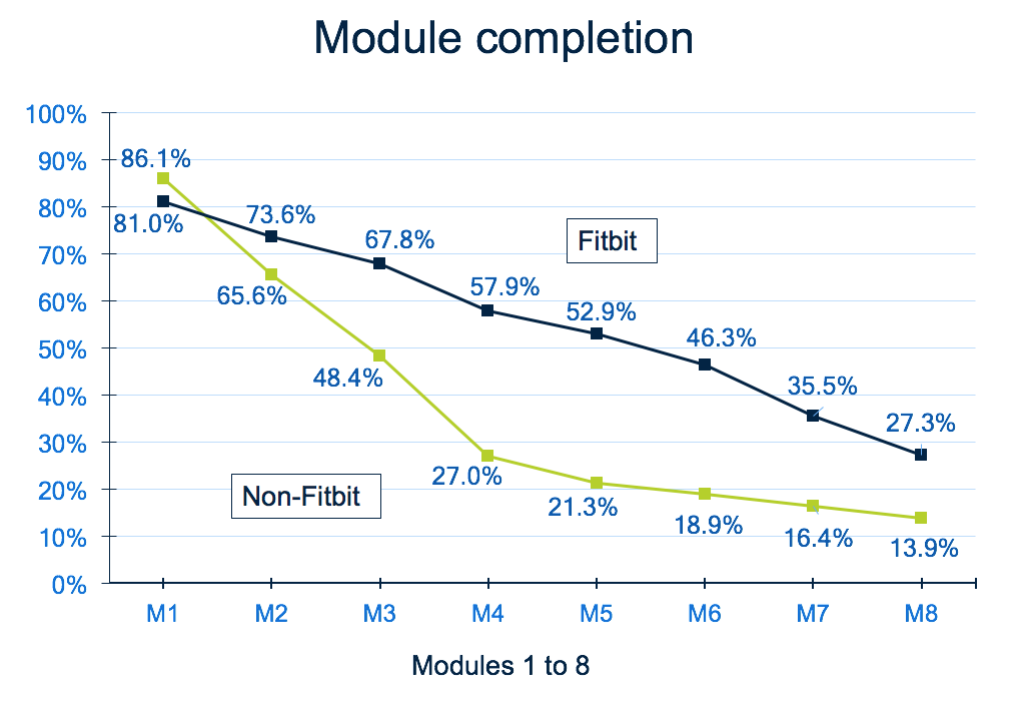
Average module completion for the Fitbit and non-Fitbit group for each of the 8 available modules.

Figure 3 demonstrates how much exposure participants had to the intervention content. A higher percentage of participants in the Fitbit group completed each module except the first one. Double the proportion of participants completed the final module in the Fitbit group compared with the non-Fitbit group (27.3% vs 13.9%). On average, non-Fitbit group participants completed 2.9 (SD 2.5) modules and Fitbit group participants completed 4.4 (SD 3.1) modules.

**Table 3:** Physical activity intervention acceptability, website use, and Fitbit use.

Acceptability and usability questions		Non-Fitbit (n=46)	Fitbit (n=78)	*P* value^a^
**Advice acceptability (% agreed or agreed strongly)^b^**				
	Did you view all the advice	45.7	70.5	.16
	There were too many questions to access the advice	30.5	16.7	.02
	I changed my opinion about being active	28.3	46.1	.15
	The tailored advice was credible	80.7	87.0	.36
	The advice taught me something new about my physical activity	41.3	65.4	.03
	Too much advice was provided per module	15.2	12.8	.44
	The tailored advice helped me reach my goals	41.3	51.3	.74
	I shared the advice with others	2.2	19.2	.006
**Website usability (% agreed or agreed strongly)**				
	I want to continue to use the website	48.1	81.0	.003
	The website is easy to use	67.3	82.3	.02
	I like the presentation of the website (layout, colors)	57.7	68.3	.48
	I used the website once per week or more	50.0	71.0	<.001
**Fitbit usability^c^** **(% agreed or agreed strongly)**				
	The Fitbit improves the value of the tailored advice	—^d^	74.4	—
	The Fitbit improves the credibility of the tailored advice	—	67.9	—
	The Fitbit improves the personal relevance of the tailored advice	—	76.9	—
	The Fitbit improves the user-friendliness of the tailored advice	—	69.3	—
	It was easy to sync data between Fitbit and the intervention website	—	85.9	—
	I wore the Fitbit every day during the study	—	73.1	—
	The Fitbit helps me to increase my physical activity	—	83.5	—
	I would like to continue using the Fitbit	—	91.2	—
	The Fitbit is easy to use	—	96.2	—
	The Fitbit is comfortable to wear	—	83.5	—

^a^The *P* values reported are the outcomes of *t* tests.

^b^All questions were scored on a 5-point Likert scale, only the sum of participants who *Agreed* or *Strongly Agreed* with each statement is presented in the table.

^c^Only participants in the Fitbit group were asked questions about Fitbit use.

^d^Not applicable.

## Discussion

### Main Outcomes

The main aim of this study was to examine whether integrating a Fitbit activity tracker into a computer-tailored physical activity intervention increased the effectiveness of the intervention. The study findings clearly support the integration of activity trackers into a Web-based physical activity intervention that provides participants with personalized advice. Total physical activity increased more than twice as much in the Fitbit group, compared with the non-Fitbit group, and moderate-to-vigorous physical activity increased nearly 3 times as much at 3 months. The lack of significant interaction effects at 1 month may be explained by participants not having received all intervention content at this stage. These findings indicate that it takes some time to change behavior, and physical activity levels were still increasing at that point in time (see Figure 2).

To date, only a few other studies have examined the use of activity trackers (ie, mostly traditional pedometers) in combination with computer-tailored advice [10,11,31]. However, none of these trials directly compared the effectiveness of a computer-tailored intervention with and without activity trackers. For example, Compernolle et al [11] demonstrated the effectiveness of step-based computer-tailored advice that used pedometers but compared this with a no intervention control group. Another study by De Cocker et al [10] compared pedometer-based computer-tailored advice with a pedometer- only group; although the group that also received the tailored advice intervention increased their activity somewhat more than the pedometer-only group, the difference was not significant. Finally, Slootmaker et al [31,32] compared activity tracker–based physical activity advice with a usual care control group and did not see improvements in physical activity in either groups. Although innovative at the time (before the proliferation of smartphone), this study may have been ahead of its time, and the acceptability and user-friendliness of the technology may have been low. The use of smartphones and advanced activity trackers is now commonplace, and the technology is generally well designed and accepted, which may explain the better results in our study. This is confirmed by the strong acceptability outcomes observed in this study. All components of the intervention (advice acceptability, website usability, and Fitbit usability) were rated more highly in the Fitbit group compared with the non-Fitbit group. Remarkably, even the design of the intervention was rated higher in the Fitbit group, despite being identical across groups. The syncing of Fitbit data also received high ratings, despite first having to sync data with the Fitbit platform (this can happen automatically depending on app and phone settings) before being able to sync with the computer-tailored intervention. The impact of the Fitbit integration is also demonstrated in terms of module completion, with twice as many participants completing all computer-tailored modules in the Fitbit group.

Although the intervention did not focus on reducing sitting time (nor did the Fitbit buzz as a prompt for prolonged sitting), substantial reductions in sitting time were observed; a significant time effect at 3 months was found for the Fitbit group, which reduced sitting by almost 12 hours per week. Many other physical activity interventions have also examined the impact on sitting time [11,33,34], and most of these studies show little to no effects on sitting time. Similarly, although the overall intervention did not focus on reducing weight nor included a diet component, substantial BMI reductions were found, with significant time effects in both groups. However, weight loss was the most popular motivation among participants for becoming more active, and a large proportion of participants did select this option (37.1%, data not reported in the Results section). For these participants only, the personalized physical activity advice they received incorporated a weight loss focus though recommending higher activity levels (no dietary advice was provided). Nevertheless, this finding is remarkable as weight loss interventions without a dietary component are often not very effective [35,36].

### Strengths and Limitations

Despite the significant findings and the novelty of the study, several study limitations should be noted; as such, the study findings should be interpreted with some caution. First, the study did not have a control group or a tracker-only group; it is possible that outcomes in the Fitbit group are because of the Fitbit itself, and not because of the combined intervention. A more robust study design (including a *Fitbit-only* group) is needed to clarify this and disentangle these effects. On the other hand, higher website usability and acceptability in the Fitbit group suggests the computer-tailored website was genuinely contributing to the increase in physical activity, as participants could have chosen to only use the Fitbit and ignore the computer-tailored website, but rather they used it more than participants who did not receive a Fitbit. Second, the intervention groups were small and dropout was high. However, the posthoc power calculations demonstrated sufficient power to detect significant between-group differences for total physical activity (89.3%) and moderate-to-vigorous physical activity (83.7%) at the 3-month time point. The total lack of face-to-face interaction with participants (thus, low accountability), may have contributed to the high levels of dropout [37,38]. High dropout rates are common in Web-based interventions [39,40]. It was nevertheless interesting to observe that just providing participants with a Fitbit significantly increased retention. Many intervention studies have found higher dropout in intervention groups (or higher intensity intervention groups) compared with control groups because of the additional burden of actively participating and trying to improve health behavior [15]; this did not apply to our study. Third, although the Fitbit objectively assessed physical activity, we were not able to use Fitbit data to assess change over time as only 1 intervention group was provided with a Fitbit. Budgetary constraints meant we had to rely on a self-report measure to assess change over time, and although the Active Australia Survey has demonstrated it can detect change over time [28], the findings should be interpreted with caution. As the introduction points out, self-report physical activity measures are prone to overreporting [9]; however, in theory, the measurement error should be consistent across groups, so it is likely that the difference between groups is real, but the magnitude of the outcomes is less certain. Fourth, there was no longer-term follow-up to assess changes in behavioral outcomes. The 3-month assessment was immediately after the end of the intervention delivery, so behavior change maintenance effects and differences between groups could not be tested. Maintenance of physical activity improvements has been very difficult to achieve, with the majority of studies showing declines in activity levels after the intervention has finished [41,42]. Finally, although the accuracy and validity of commercial consumer-level activity trackers are high, there is room for improvement [12]. As such, in a small number of participants, the personalized advice generated using Fitbits may still have been somewhat inaccurate and indicated they were meeting guidelines when they were not in reality. Therefore, manufacturers are encouraged to continue to improve the quality of the devices, and researchers are encouraged to continue to assess their accuracy in validity studies.

### Conclusions

In conclusion, integrating physical activity trackers into a Web-based computer-tailored intervention significantly increased intervention effectiveness in overweight or obese participants. Due to the technology-based nature of this intervention, it is possible to reach a large number of people at an acceptable cost and improve their physical activity behavior. As such, the potential of combining advanced activity trackers with sophisticated computer-tailored interventions is large. However, given the study limitations, follow-up studies with more robust designs (objective outcome measures and longer-term follow-up including control and tracker-only groups) are needed to confirm these outcomes.

## Appendix

Multimedia Appendix 1 CONSORT‐EHEALTH checklist (V 1.6.1).

## Abbreviations

BMI: body mass index
CQUniversity: Central Queensland University
PAR-Q: physical activity readiness questionnaire
